# Parametric Analysis on the Circular CFST Column and RBS Steel Beam Joints

**DOI:** 10.3390/ma12091535

**Published:** 2019-05-10

**Authors:** Rui Li, Yang Yu, Bijan Samali, Chengyu Li

**Affiliations:** 1 Centre for Infrastructure Engineering, Western Sydney University, Penrith, NSW 2751, Australia; r.li2@westernsydney.edu.au; 2 Urban Construction College, Wuhan University of Science and Technology, Wuhan 430065, China; lee_chengyu@163.com

**Keywords:** finite element analysis, reduced beam section (RBS), concrete-filled steel tubular (CFST) column, diaphragm-through, analysis of geometric parameters

## Abstract

This research analyzes the results of parametric studies of concrete-filled steel tubular (CFST) columns to the reduced beam section (RBS) beam joint with through diaphragm, using ANSYS. Several indices that are able to characterize the cyclic behavior of the composite joints are investigated, including the stiffness degradation, strength deterioration, stress distribution, and energy dissipation capacity. Four main model parameters, including the distance from the diaphragm edge to the cut start, the cut length, the cut depth, and inner diameter of through diaphragm, are analyzed via comparative studies to investigate their impacts on seismic properties of the joint. Finally, the orthogonal experiment is conducted to study the effects of these parameters on the strength and energy dissipation, the results of which are capable of achieving optimal seismic behavior of the joints.

## 1. Introduction

A concrete-filled steel tubular (CFST) column with through diaphragm joints are manufactured as through diaphragm welds to steel tube and then welds to steel beams [1,2,3]. This type of joint does not use electro-slag welding, and the through diaphragm can transfer the tension and shear of steel beam to the concrete core directly, which effectively restrains the steel tubes in terms of local buckling. Nowadays, the CFST column with through diaphragm joints have already been used in medium- and low-rise building [4,5,6].

In the past few years, a limited number of tests and finite element analyses (FEA) were conducted to study steel beam connected to CFST columns by through diaphragm. Nishiyama et al. studied the relationship between loading and deformation as well as ultimate strength of CFST beam-to-column sub-assemblies, according to the results of nine through diaphragm experimental specimens with various material strength, structural configurations, and load scenarios [7]. From this study, a phenomenological model for characterizing the restoring force of the shear panel in the sub-assemblies was presented, which can repeat the results from experimental testing, with high accuracies. Rong et al. studied the shear performance of a CFST column with T-shape through diaphragm joints under cyclic loads [8]. The experimental results demonstrated that the shear performance of the designed beam-column joint heavily relies on steel tube and core concrete, even though the flange has a slight influence on shear performance. Kanatani et al. studied the mechanical properties of concrete-filled RHS column to through diaphragm connection under cyclic loads [9]. The result shows that this type of connection has excellent strength and stiffness. Chen et al. conducted several groups of cyclic loading test on the beam-column joint, with crossing diaphragm [10]. The factors taken into account in the test include, the steel circular and rectangular tubes, crossing diaphragm and inner diaphragm, the axial load level on column, and the extrusion of diaphragm. It was shown that, such a type of joint satisfies the requirement indicated in existing seismic design standards. Finite element analyses and static tensile load tests were conducted by Rong et al. for studying mechanical performance and failure mode of CFST square column diaphragm-through joints [11]. They observed that tensile stress from steel beam flange was mainly absorbed by square steel tube and diaphragm.

**Figure 1:**
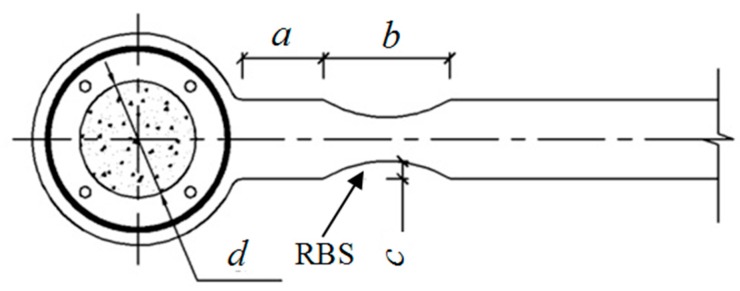
The detail of the innovative joint (RBS, reduced beam section).

A reduced beam section (RBS) is a common method to make the beams weaker than columns. Meanwhile, RBS primarily yield and buckle under seismic loadings, and improves the flexural and inelastic deformation demands in the column. An innovative type of joint, circular CFST column welds to RBS beam with through diaphragm was proposed and studied by Li and Li [12]. It concludes that joint with RBS exhibits a better seismic performance than the joint without RBS. This study will attempt to conduct a further parametric investigation of this composite joint. Figure 1 shows the schematic of the proposed joint. First of all, a finite element (FE) model is built-up using ANSYS (Canonsburg, PN, USA), the effectiveness of which is validated, based on experimental data from existing literatures. Then, several key parameters of the developed finite element (FE) model are investigated to evaluate their influences on seismic performance of the joint. The outcomes of this study can provide the theoretical guidance for civil engineers to design the circular CFST column and RBS steel beam joint for practical applications. The main objectives in this paper can be summarized as follows: (i) To conduct geometrically parametric studies using FE analysis, in which the parameters consist of the distance from the diaphragm edge to the cut start (*a*), the cut length (*b*), the cut depth, (*c*) and internal diameter of through diaphragm (*d*); (ii) to investigate the importance order of four parameters via their influences on seismic properties of this innovative joint and propose optimal design solution using orthogonal design.

## 2. Development of Finite Element Model

### 2.1. Numerical Model Design

**Figure 2:**
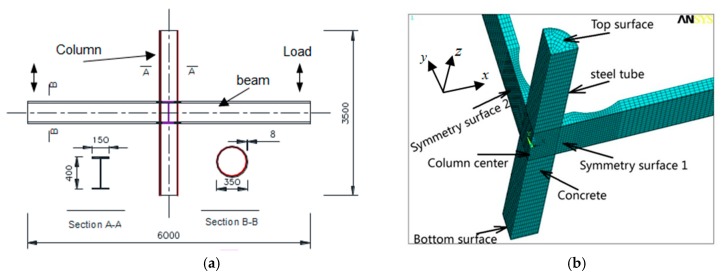
Joint dimensions and boundary conditions. (**a**) Configuration of joints, (**b**) Boundary conditions.

In this study, the ANSYS software is employed to conduct the FE analysis, in which the calculation considers both materials and geometric nonlinearities and the solution is obtained using Newton-Raphson incremental iteration method. A schematic view of a FE model for CFST columns to reduced beam section (RBS) steel beam connection is presented in Figure 2.

In the designed FE models, a steel frame, with bay length of 6.0 m and the height of 3.5 m, is simulated. Besides, an H-shape beam, with section of flange width of 150 mm by depth of 450 mm, is employed as well. The thickness values of flange and web are 12 mm, and 8 mm, respectively. The CFST column with circular steel tube of wall thickness of 8 mm and diameter of 350 is utilized. Federal Emergency Management Agency (FEMA) proposed a recommended seismic design criterion of the RBS in 2000 [13]. Although this recommendation is based on the joint of universal column to H beam, the geometric dimensions of the RBS can be preliminarily designed by referencing to this criterion.

### 2.2. Element Types and Meshes

To select proper elements to characterize the seismic performance of the joints, different types of elements are employed to obtain the best result. It is found that a solid element is prone to be more useful in simulating the steel beam as well as concrete. To simulate the concrete core, the SOLID65 with eight-node brick element is employed; to simulate the steel tube and beam, the SOLID45 is adopted. The interface between concrete and steel is simulated between two matching surfaces (using targe 170 and conta 173), which can be separated from each, other even though it is not allowed for penetration. Contacts in tangential and normal directions are defined between in-filled concrete and steel tube. In the tangential direction, the Mohr-Coulomb friction contact is employed, while in the normal direction, the hard contact type is utilized. In this analysis, tangential friction coefficient between in-filled concrete and steel tube is set to 0.3.

In order to guarantee the appropriate element shape, the structured mesh method is adopted, whereby different sizes of grid are tried to obtain the optimal grid size. Through a large number of trials, it can be found that a mesh size of 1: 2: 2 (depth: width: length) can obtain optimal performance with best calculation efficiency for both concrete and steel elements [14]. Figure 2 provides the typical joint meshes.

### 2.3. Boundary Condition and Loading Type

Except for the displacement of the top surface in Z direction, both bottom and top surfaces of the CFST column are fixed against all the DOFs. Figure 2b shows the quarter of the established joint in ANSYS because of structural symmetry. The nodes on symmetric surface 1 and surface 2 are specified symmetry degree-of-freedom constraints in two directions X, and Y, respectively. The nodes on the center line of the column are avoided from displacement in these two directions. Moreover, other nodes have not had any constraints in any direction.

**Figure 3:**
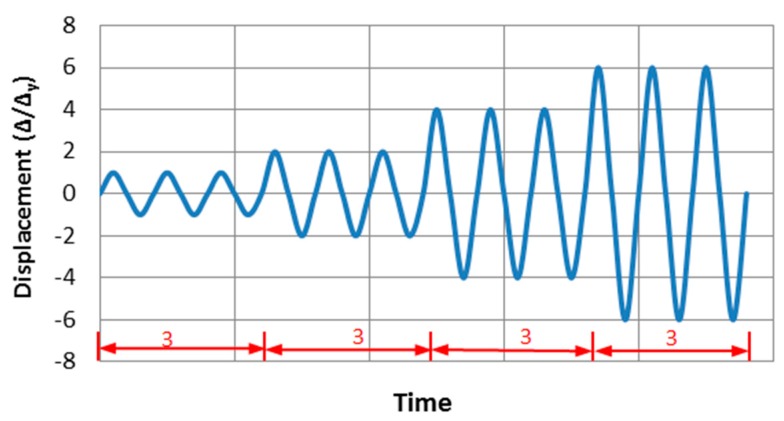
Cyclic loading protocol.

There are two types of loading in this study, i.e., (1) monotonic loading: Imposing the displacement load of 50 mm on both tips of the beam along the *z*-axis direction (Figure 2a), and this type of loading is divided into ten load sub-steps, and (2) cyclic loadings: The load order is regulated by the yield displacement (*∆*_y_), the value of *∆*_y_ is 7.4mm, which is obtained from FE results, suffered to monotonic loading. The beam end is loaded with three full cycles, as shown in Figure 3. Initially, the axial loading is applied to the top of the column, and then the monotonic loading or cyclic loadings are simultaneously applied on the both beam tips. The nonlinear FE models are solved using Newton–Raphson equilibrium iteration approach.

### 2.4. Material Modeling of Steel and Concrete

**Figure 4:**
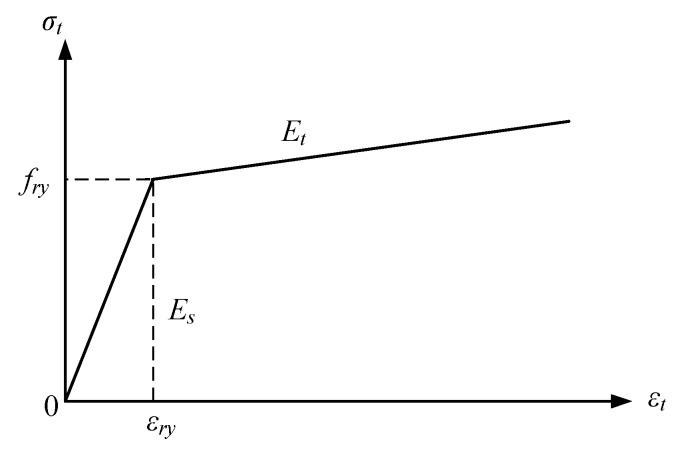
Bilinear stress–strain relation model.

In the FE analysis, the nonlinear kinematic hardening rule with the Von Mises yield criterion is employed. To simulate the steel beam and tube, bilinear model for stress-strain is utilized, as shown in Figure 4. The Poisson’s ratio and yield strength are 0.28, and 350 MPa, respectively. Elastic modulus *E_s_* is 2.06 × 10^5^ MPa, and hardening modulus is one-tenth of elastic modulus. The cylindrical compressive strength of concrete is 40 MPa.

Han et al. [15] developed an accurate and practical model for in-filled core concrete in the CFST column, which has been used by other studies [14,16,17]. The equation is as follows.

(1) $$y=\{\begin{array}{c} 2x-x^{2}\;\;\;x\leq 1\\ \frac{x}{\beta_{a}{\left(x-1\right)}^{2}+x}\;\;\xi <1.12;\;x>1\\ 1+q\left(x^{0.1\xi }-1\right)\;\xi \geq 1.12;\;x>1 \end{array}$$

(2) $$x=\frac{\varepsilon }{\varepsilon_{0}};\;\;y=\frac{\sigma }{\sigma_{0}}$$

(3) $$\sigma_{0}=\left[1+\left(-0.0054\xi^{2}+0.4\xi \right){\left(\frac{24}{f_{c}^{\prime }}\right)}^{0.45}\right]f_{c}^{\prime }$$

(4) $$\varepsilon_{0}=\varepsilon_{cc}+\left[1400+800\left(\frac{f_{c}^{\prime }}{24}-1\right)\right]\xi^{0.2}$$

(5) $$\varepsilon_{cc}=1300+12.5f_{c}^{\prime }$$

(6) $$q=\frac{\xi^{0.745}}{2+\xi }$$

(7) $$\beta_{a}={\left(2.36\times 10^{-5}\right)}^{\left[0.25+{\left(\xi -0.5\right)}^{7}\right]}\sqrt{f_{c}^{\prime }}\times 3.51\times 10^{-4}$$

(8) $$\xi =\frac{A_{s}f_{y}}{A_{c}f_{c}^{\prime }}$$

where $f_{c}^{\prime }$ denotes cylindrical compressive strength of concrete; *f_y_* represents the steel yield strength; *A_s_* denotes the area of steel in the CFST cross-section; *A_c_* denotes the area of concrete in the CFST cross-section; *ξ* denotes the confinement factor. 

This paper will use this material model to simulate the core concrete, and its Poisson’s ratio is set as 0.2. 

### 2.5. Model Verifications

**Figure 5:**
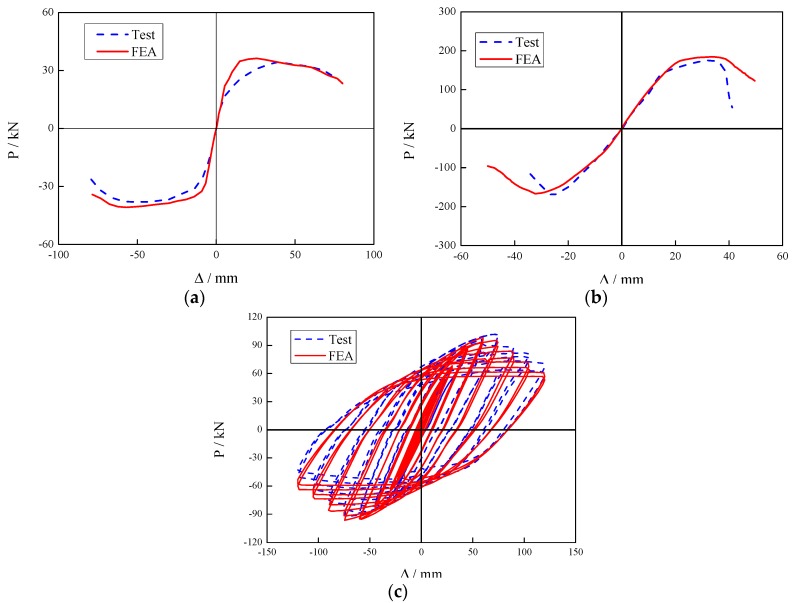
Comparison between the test results and finite element analyses (FEA) predictions (P: Load applied to the beam end, ∆: displacement at the beam end). (**a**) SJ-32; (**b**) CFRTJ-3; (**c**) CJ-22.

In order to obtain accurate results, the proposed method of developing the FE models was verified by comparing the existing test data published in the literature. Wang et al. [18] conducted experimental tests on the composite joints, composed of steel beam and concrete filled SHS column, with stiffening ring, and the lateral loadings were applied on the column top. Nie at el. [14] investigated the joints, consisting of steel-concrete composite beams and CFST square columns under monotonic loading. Experimental research on steel beam to CFST hollow section column, using stiffening rings were studied by Wang et al. [19]. The lateral loadings of the experimental tests from the references [18], [14] and [19] are applied on the beam tips for the numerical model verification. Figure 5a–c shows the FEA results, compared to the test data mentioned above. It is indicated that the test and numerical results are in good agreement.

## 3. Numerical Results

Four geometric parameters could affect the behaviors of the circular CFST column and RBS steel beam joints, including distance from the diaphragm fringe to the cut start (*a*), cut length (*b*), cut depth (*c*), and internal diameter of through diaphragm (*d*). The ranges of the four parameters used in this investigation are listed in Table 1. Model RR is defined as the reference joint.

**Figure 6:**
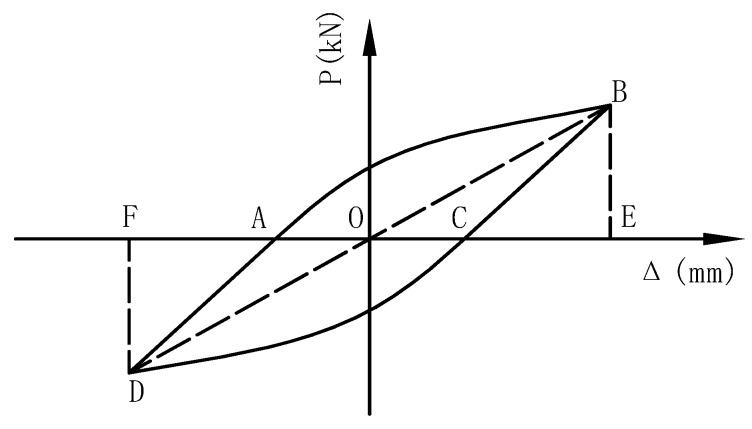
Calculation of equivalent dissipation coefficient.

The load (*P*)–displacement (*Δ*) relationship of joints under monotonic loading, the Von Mises stress distribution along beam length and welding seam, *P*–*Δ* envelope curves, index of looped rigidity degradation and equivalent dissipation coefficient are discussed in this study. Two useful calculation formulas are presented as follows:

(1) The equivalent dissipation coefficient (*β*) could be employed as an index to represent the model energy dissipation performance [20]. This mathematical expression is given in Equation (9) and also illustrated in Figure 6:
  (9) $$\beta =\frac{1}{2\pi }\frac{S_{ABCDA}}{S_{OBE}+S_{ODF}}$$
  where *S_ABCDA_*, *S_OBE_* and *S_ODF_* denote the areas bounded by corresponding points.
(2) The stiffness reduction of models subjected to cyclic loads could be estimated using index of looped rigidity degradation (*K*) [21], which is defined by:
  (10) $$K=\frac{K_{i}}{K_{1}}$$
  (11) $$K_{i}=\frac{\sum_{i=1}^{n}P_{j}^{i}}{\sum_{i=1}^{n}\Delta_{j}^{i}}$$
  where *K* denotes the index of looped rigidity degradation; *K_i_* denotes the *i*th cyclic stiffness; *K*_1_ denotes the unload stiffness; $\Delta_{j}^{i}$ represents the peak displacement of the *i*th cycle; $P_{j}^{i}$ denotes the peak load of the *i*th cycle and *n* denotes the times of loading cycle.

### 3.1. Influence of the Range from Diaphragm Fringe to Cut Start (a)

**Figure 7:**
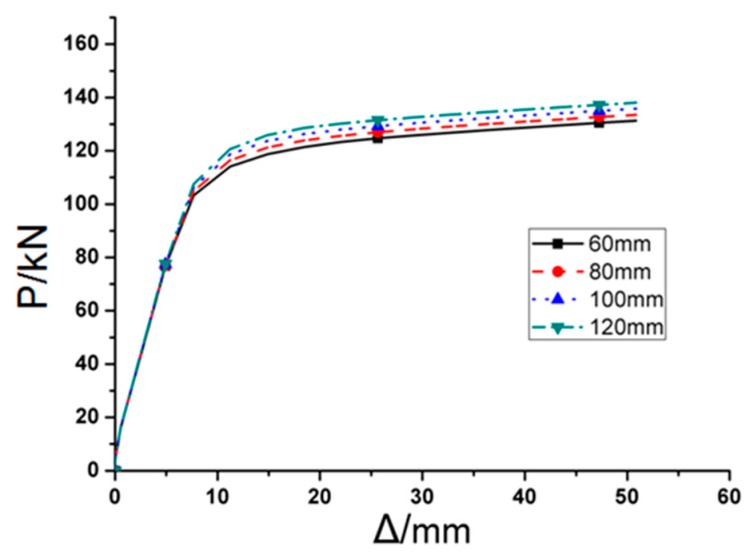
The load-displacement curves of RA series finite element (FE) joint models under monotonous load.

RA series FE models, included RA1, RA2, RA3, and RR, had variable *a* and the rest three parameters *b*, *c* and *d* kept constant. Figure 7 depicts the load-displacement (*P–Δ)* curves of RA series FE models under monotonic load, it is clearly observed that initial stiffness of these four joints are almost same, which can be reflected by overlapping portion in the *P*–*Δ* curves.

**Figure 8:**
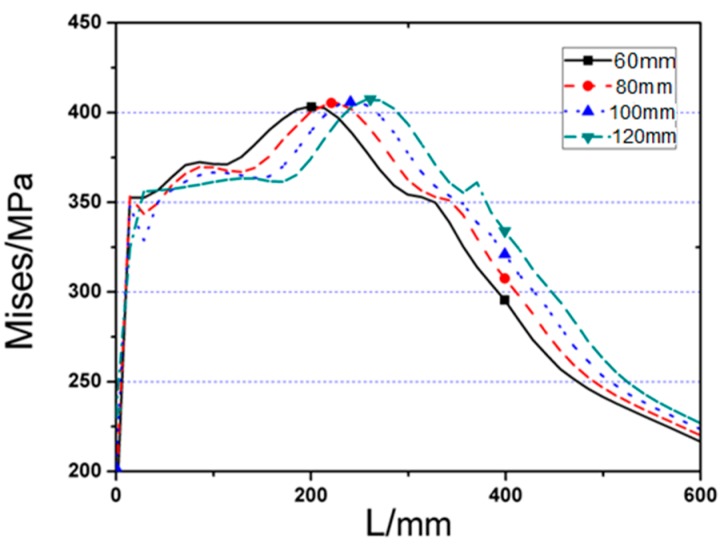
The Von Mises stress distribution along beam length (start from steel tube) of RA series FE joint models.

**Figure 9:**
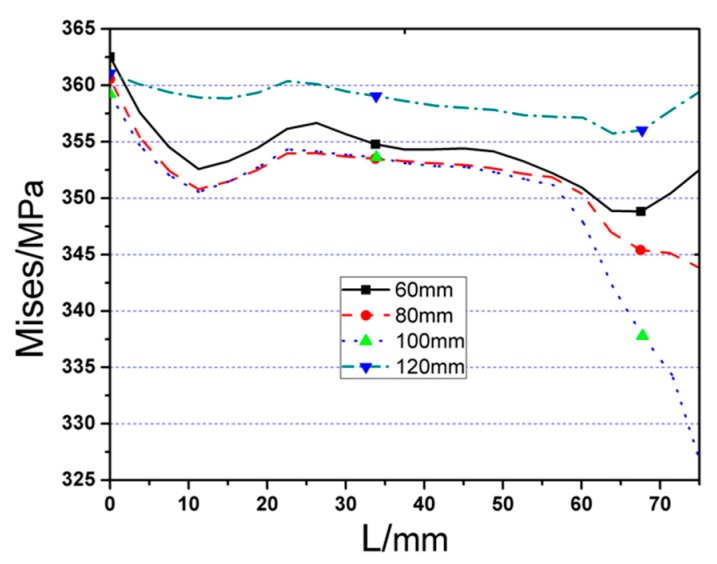
The Von Mises stress distribution along welding seam length (start from edge to middle of welding seam) of RA series FE joint models.

**Figure 10:**
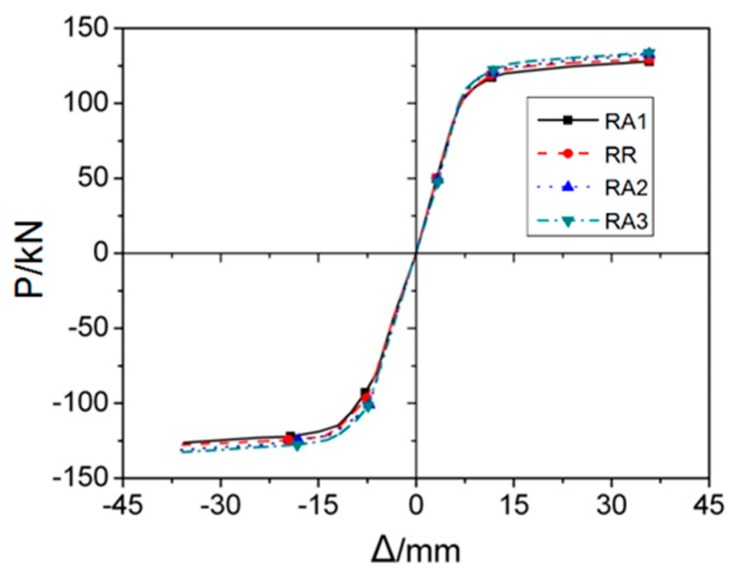
The skeleton curves of RA series FE models under cyclic loading.

As the cut length *a* increases, joint yield strength and ultimate bearing capability are both moderately improved. Furthermore, changing the value of *a* can affect the location of the plastic hinge and stress distribution on welding seam. Figure 8 and Figure 9 describe the Von Mises stress distributions along the beam length, and welding seam, respectively, and the value of maximum Von Mises stresses of RA series models and the range from steel tube to plastic hinge location are listed in Table 1. From the comparison results, it can be found that:

(1) Maximum Von Mises stresses of four FE models are approximately equal, which indicates that the strength of the joint is hardly changed with the variation of parameter *a*.
(2) All maximum Von Mises stresses are located on the middle of the RBS, which is capable of preventing weld from brittle failure.
(3) It is clearly observed that maximum Von Mises stresses, along the length of welding seam, are located on the welding seam edge, as shown in Figure 10, and the stress decreases with the increase of cut length. However, once the value of *a* is bigger than 0.65*b_f_*, where *b_f_* represents beam flange width, the stress on welding seam increase again. The main reason contributing to this phenomenon is the effect that RBS becomes inconsiderable if the RBS moves away from the weld, thus the plastic hinge could not be developed at RBS.

**Figure 11:**
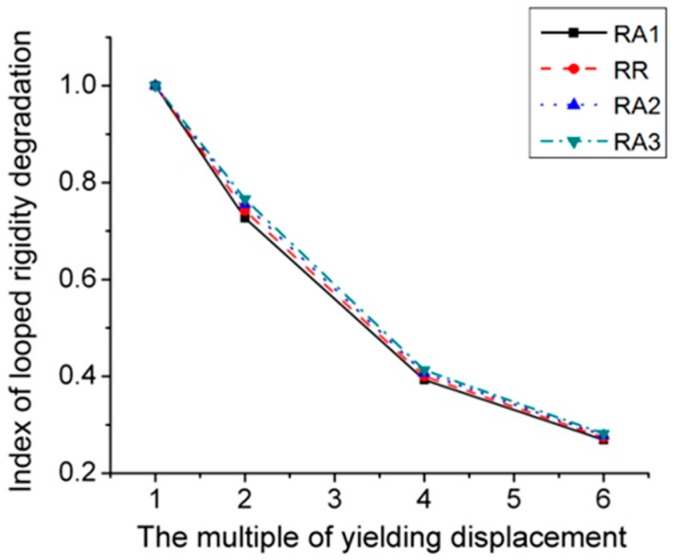
Index of looped rigidity degradation curves of RA series joints.

Figure 10 and Figure 11 illustrate the skeleton and rigidity degradation curves of four RA series joints subjected to cyclic loads, respectively. It can be seen that the higher the value of *a*, the better performance of joint in terms of yielding strength, stiffness, ultimate strength and energy dissipation capacity. The main reason contributing to this result is that the stiffness of joint panel zone is reduced with RBS moving close to column. Furthermore, from the Figure 11, it is noted that the stiffness degradation decreases slowly with the increase of loading cycles. 

Overall, the distance from the welding seam to the start of the cut (*a*) is expected as far as possible, which can prevent the RBS from thermal degradation during welding, and avoid stiffness and bearing capacity decreasing resulted by prematurely yielding of RBS. In addition, as mentioned above, joint strength enhances with the increase of *a*. Nevertheless, once the value of *a* exceeds 0.65*b_f_*, the effect of RBS is inconsiderable. As a result, the reasonable set of dimension a for through diaphragm joint is (0.5–0.65)*b_f_*.

### 3.2. Influence of the Length of the Cut (b)

**Figure 12:**
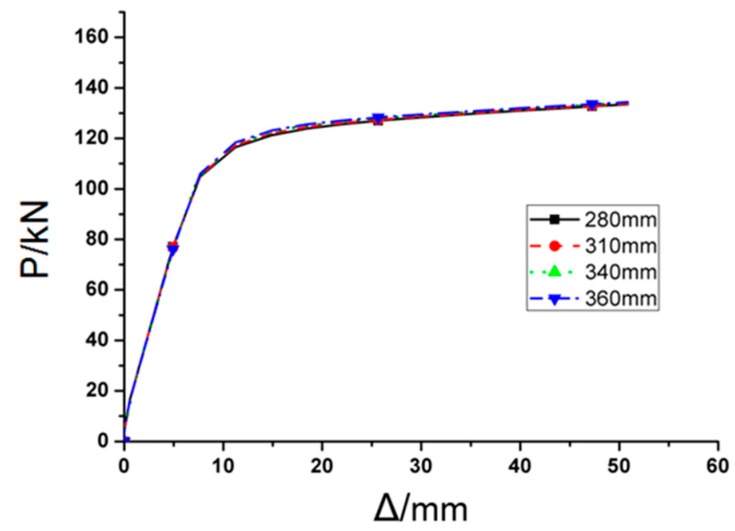
The load-displacement curves of the RB series FE joint models under monotonous loading.

For the RB series FE models (RB1, RB2, RB3, and RR), with variable value of *b*, but the rest three parameters *a*, *c,* and *d* stayed the same. Figure 12 shows the *P*–*Δ* curves of four RB series FE joint models under monotonic loads. It is clearly seen that the relationships between load and displacement of four models are almost coincident. However, there are subtle differences at the hardening stage, where the strength of the joint with larger parameter *b* is slightly better than that of the models with smaller curt length.

**Figure 13:**
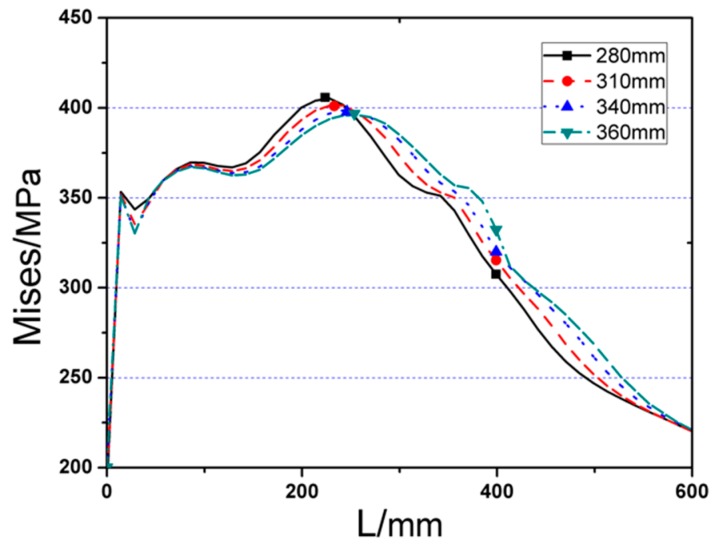
The Von Mises stress distribution along beam length (start from steel tube) of RB series FE joint models.

**Figure 14:**
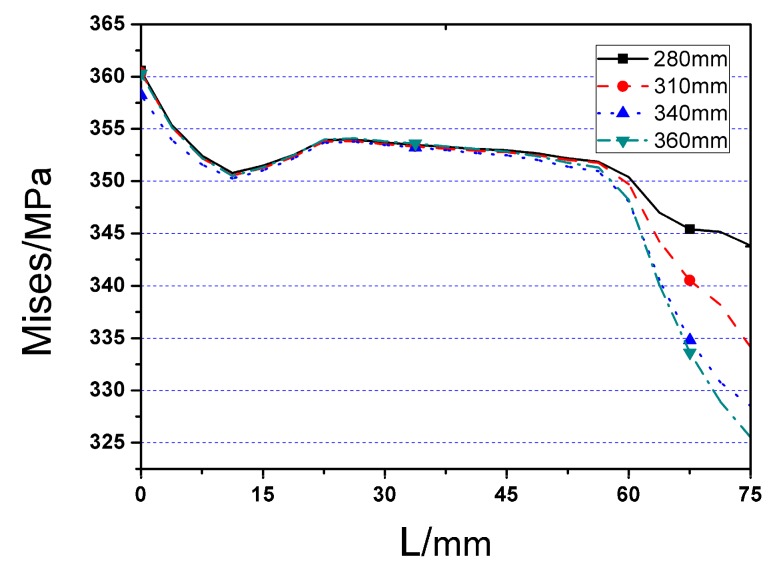
The Von Mises stress distribution along welding seam length (start from edge to middle of welding seam) of RB series FE joint models.

**Figure 15:**
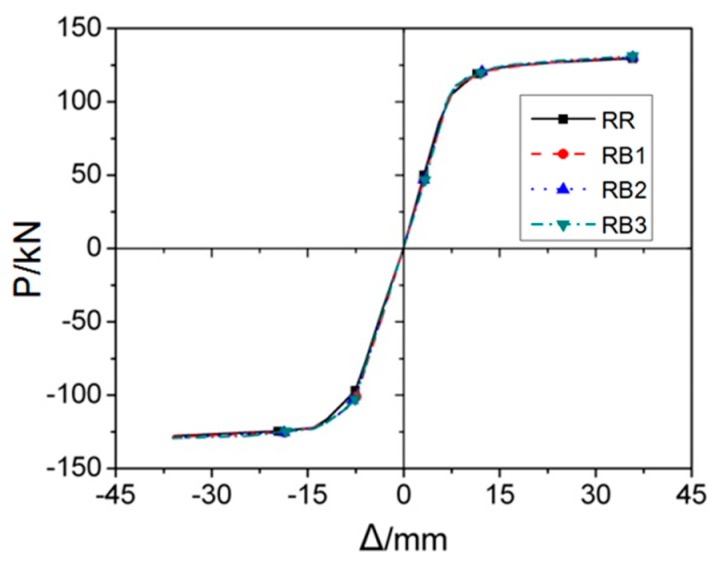
The skeleton curves of RB series FE models under cyclic loading.

The Von Mises stress distributions along beam length and welding seam length of the four RB series FE models are displayed in Figure 13 and Figure 14, respectively. The detailed values of maximum Von Mises stresses of RB series FE models and the distances from the fringe of through diaphragm to the location of plastic hinge for four cases are indicated in Table 1. From the results, it can be concluded that:

(1) Although the peak Von Mises stress happens at the weakest part of RBS for all the cases, the stress distribution of the joint, with a higher value of cut length (*b*), is more uniform, and the corresponding maximum stress is smaller as well.
(2) Using different cut length, there are not obvious variations on the maximum stress of welding seam, as illustrated in Figure 15.

**Figure 16:**
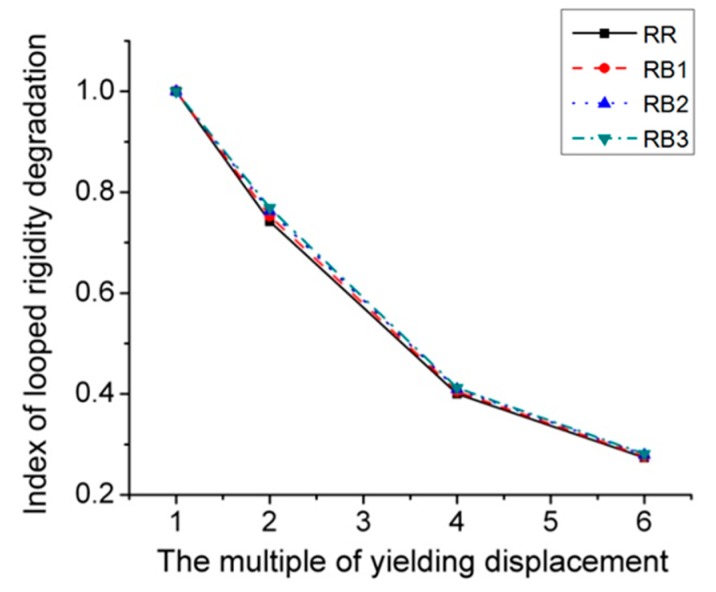
Index of looped rigidity degradation curves of RB series joints.

Figure 15 describes *P*–*Δ* skeleton curves of four RB series FE models subjected to cyclic loading, and Figure 16 provide the corresponding curves for cyclic stiffness degradation. Table 1 displays the equivalent dissipation coefficient values of RB series models, which are used to indicate the energy dissipation capacity of joints. It is clearly observed from the figures and table that there are no obvious changes in terms of initial stiffness, yield load, and bearing capacity. Even though the increase in value of parameter *b* has little influence on the joint’s stiffness degradation, it can still contribute to the slight improvement of energy dissipation performance, which can be observed in Table 1.

Consequently, the cut length (*b*) has little influence on the performance of this type of joint, and the range of cut length recommended by FEMA, *b* = (0.65 to 0.85)*d_b_*, is suitable for the CFST column to RBS steel beam joint, where *d_b_* denotes beam depth.

### 3.3. Influence of the Depth of the Cut (c)

**Figure 17:**
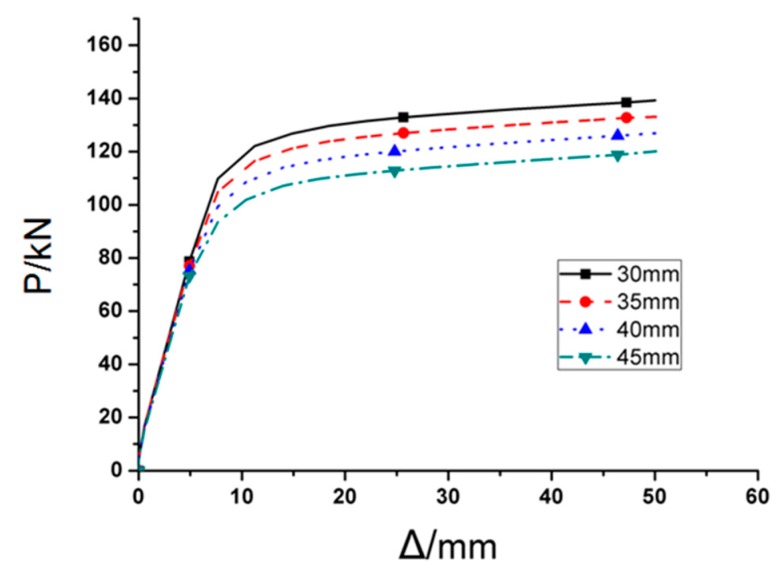
The load-displacement curve of RC series FE models under monotonous loading.

**Figure 18:**
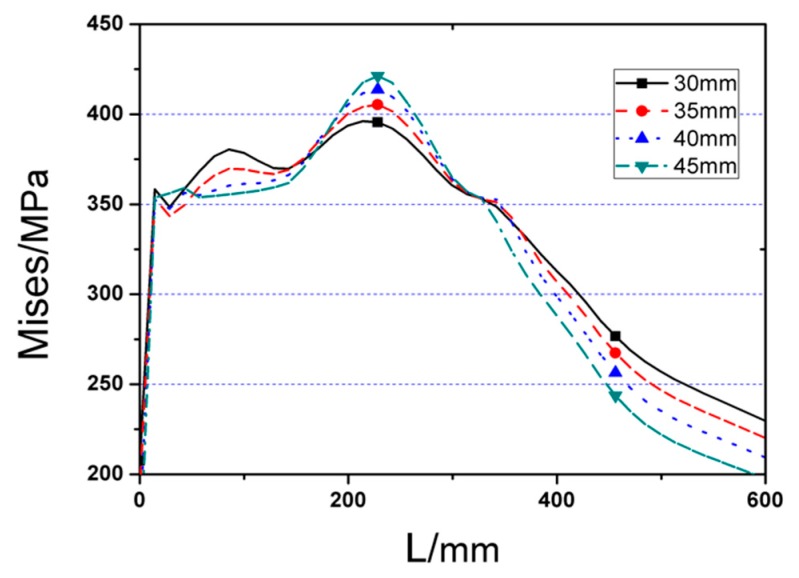
The Von Mises stress distributions along beam length (start from steel tube) of RC series FE joint models.

**Figure 19:**
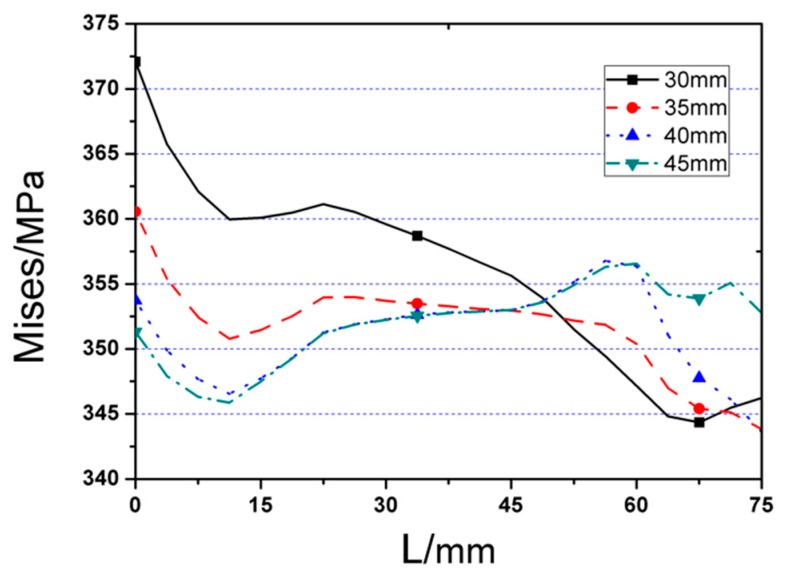
The Von Mises stress distributions along welding seam length (starting from edge to middle of welding seam) of RC series FE joint models.

Named RC1, RC2, RC3, and RR as RC series FE models. Value of *c* is the variable, while the rest three parameters *a*, *b,* and *d* are kept as the constants. Figure 17 depicts the *P*–*Δ* curves of the four RC series FE models under monotonic load, in which obvious differences are observed in terms of peak load for the joint models with different cut depths. Besides, it is noticeable that the FE joint model with deeper cut reaches yielding point earlier, and ultimate strength are dramatically lower than those with shallow cut on RBS. Increasing every 5 mm (0.033*b_f_*) in cut depth will result in 4.5% loss of ultimate strength. Figure 18 and Figure 19 present the Von Mises stress distributions along the beam and welding seam lengths of four RC series FE models, while Table 1 lists the value of maximum Von Mises stresses of RC series models and the range from steel tube to plastic hinge location. According to these results, the following conclusions could be obtained:

(1) Obviously, changing cut depth (*c*) would not affect the location of peak Von Mises stress, while the value of peak Von Mises stress declines significantly with the decrease of cut depth.
(2) Although increasing cut depth can reduce the stress on the welding seam edge, the stress on the middle of welding seam ascends gradually at the same time, even exceeding the stress of the edge when the cut depth is over 40 mm.

**Figure 20:**
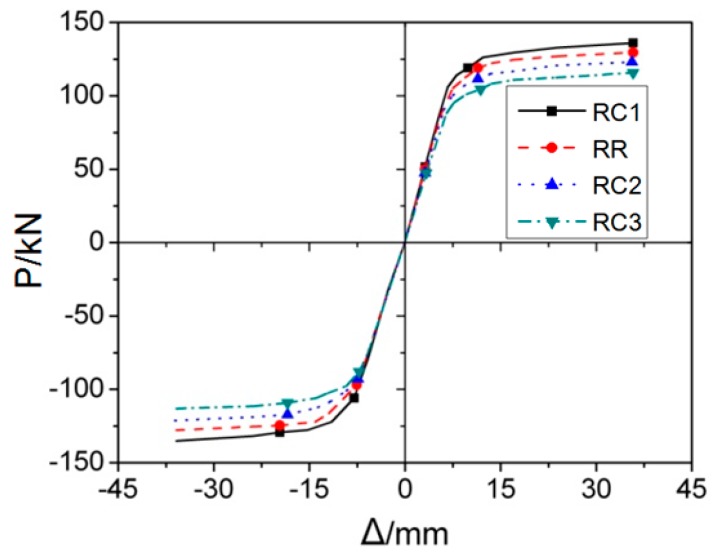
The skeleton curves of RC series FE models under cyclic loading.

**Figure 21:**
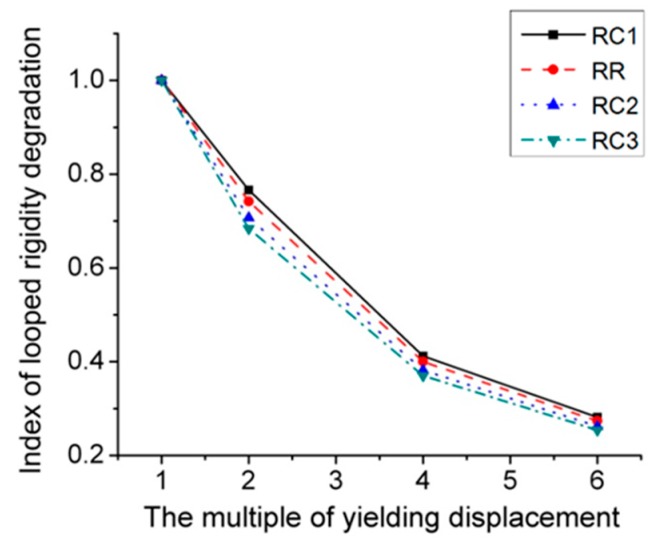
Index of looped rigidity degradation curves of RC series joints.

Then, the RC series FE models was applied to the cyclic loading and the corresponding results are illustrated in Figure 20 and Figure 21, where Figure 20 gives the *P*–*Δ* skeleton curves and Figure 21 describes the cyclic stiffness degradation curves of four models. Table 1 displays the equivalent dissipation coefficients of the joint FE models with different values of *c*, which are employed to demonstrate their energy dissipation capacities. It can be found that the cut depth has remarkable effect on the cyclic behavior of the joint. With the addition of the value of *c*, the yield strength, and ultimate strength of the joint will be decreased accordingly. However, the energy dissipation capacity of the joint keeps almost unchanged. During cyclic loading, the joint with the high value of *c* will degrade more. 

To sum up, the joint with the larger cut depth (*c*) generally has smaller stiffness, strength, and bearing capacity, and its effect is more obvious. In a certain range, the larger the value of *c*, the smaller the stress at the welding seam. However, if the cut depth exceeds its reasonable range, the stress will arrive at the welding seam before it is evenly distributed in the cross-section of the flange, which will result in the stress concentration on the middle part of the welding seam and subsequent brittle failure. Accordingly, on basis of the FE simulation results, the optimal value of *c* should be in the range of (0.2–0.25)*b_f_*.

### 3.4. Influence of the Inner Diameter of through Diaphragm (d)

**Figure 22:**
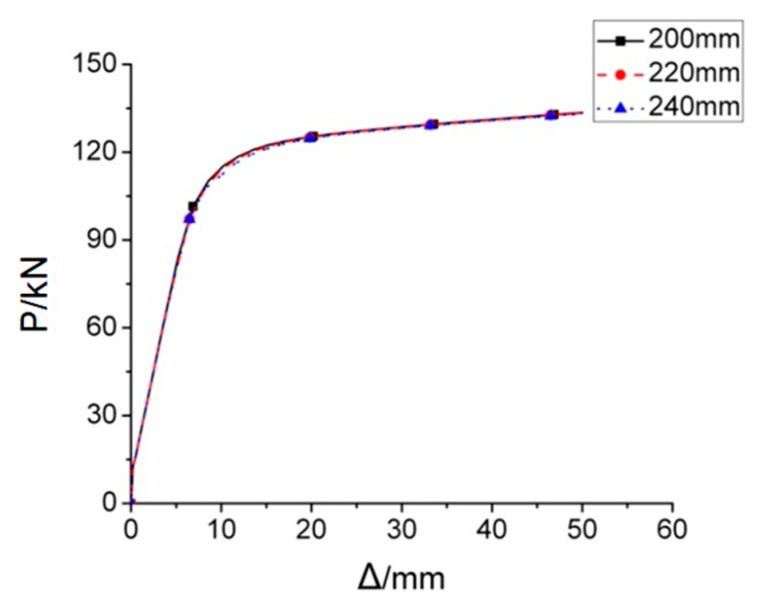
The load-displacement curves of RD series FE models under monotonous loading.

The smaller the increment of *d* is, the more accurate the range of *d* proposes. FE models with various *d*, from 160 mm to 300 mm with increment of 10 mm, were assessed. Therefore, the *P*–*Δ* curves are almost same if *d* ≤ 240 mm, while if *d* ≥ 250 mm, FE analyses won’t be completed due to un-convergence issue, only the joints with d = 200 mm, 220 mm, 240 mm, 250 mm, and 300 mm, is selected and illustrate in this paper. Figure 22 provides the load-displacement curves of FE models with different values of parameter *d* under monotonous load. It is clearly observed from the figure that three *P*–*Δ* curves are approximately in the same shape, which indicates that the inner diameter of through diaphragm, changed in a rational range, will not significantly affect the stiffness, strength, and ductility of the joint. 

**Figure 23:**
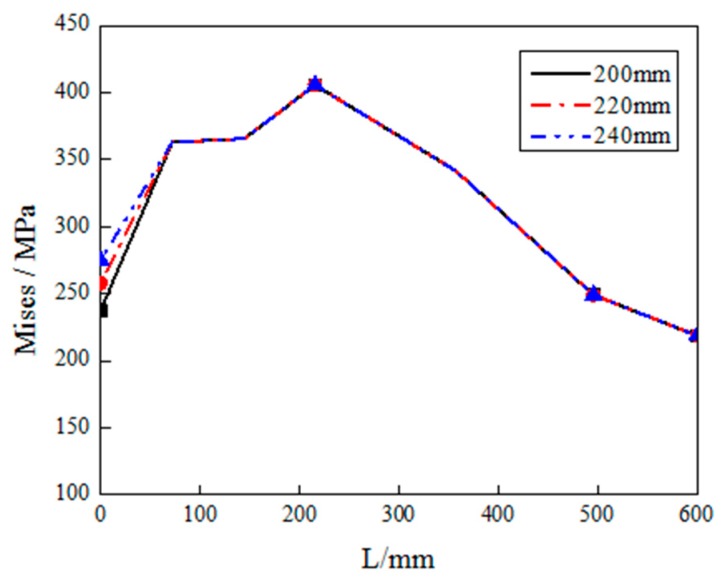
The Von Mises stress distributions along beam length (starting from steel tube) of RD series FE joint models.

**Figure 24:**
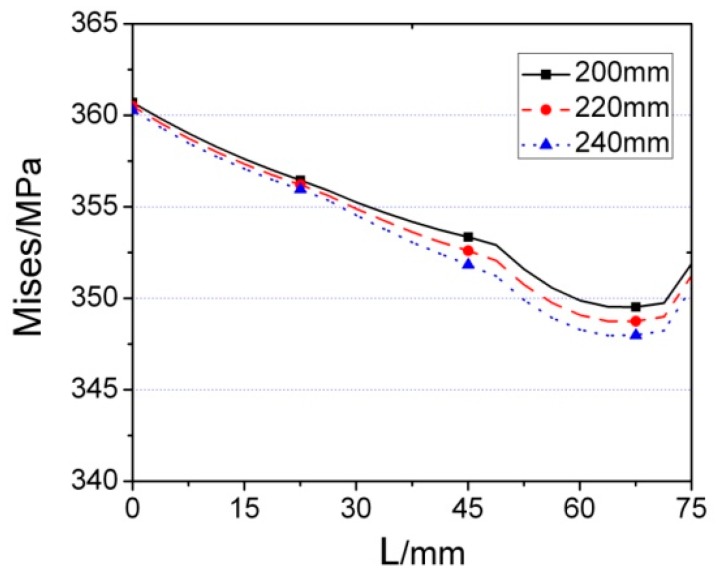
The Von Mises stress distributions along welding seam length (starting from edge to middle of welding seam) of RD series FE joint models.

In order to further study the behavior of the joint with different inner diameters under monotonic loading, the Von Mises stress distribution of RD series FE models (RD1, RD2, and RD3), along the length of beam and the welding seam, are investigated, as shown in Figure 23 and Figure 24. Although *d* has a little effect on the stress of the diaphragm, the stress distributions of the three RD series FE models on beam are the same, which demonstrates that the value of parameter *d* has no influence on the stress of the beam. In addition, the stresses along the welding seam are slightly decreased when the value of *d* is increased.

**Figure 25:**
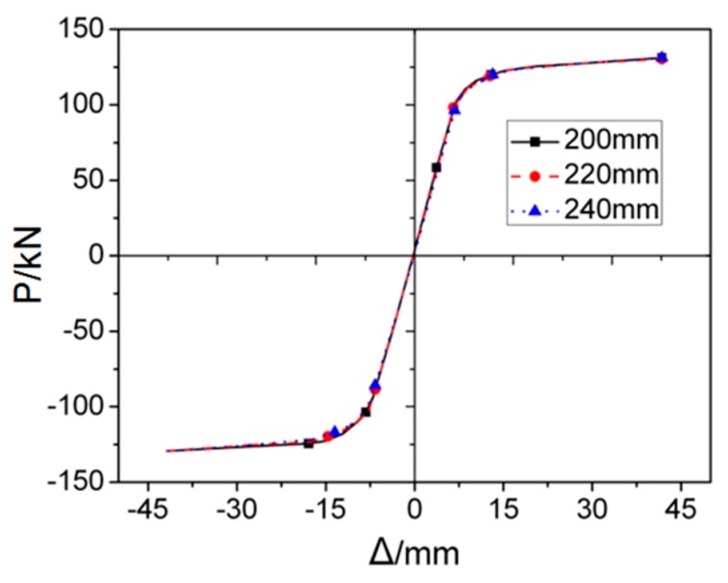
The skeleton curves of RD series FE models under cyclic loading.

**Figure 26:**
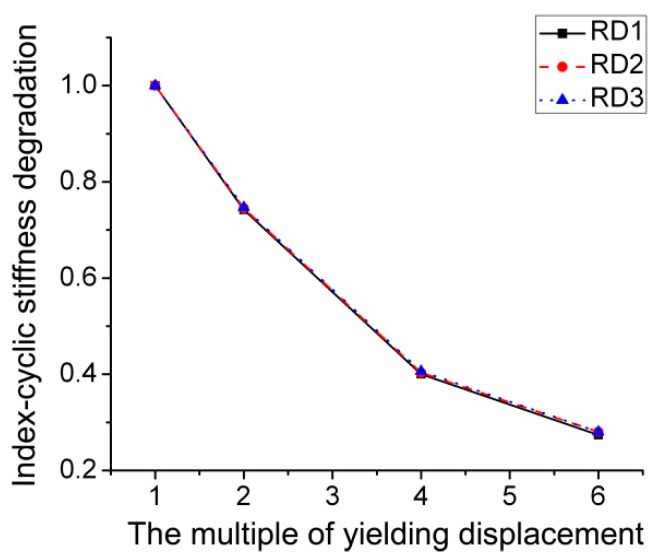
Index of looped rigidity degradation curves of RD series joints.

The skeleton curves of three RD series FE models are shown in Figure 25. It is noticeable that three curves with different values of *d* are almost coincident. Similar phenomenon can be observed in Figure 26, which signifies the influence of different inner diameter of through diaphragm on the stiffness degradation. Table 1 provides the equivalent dissipation coefficients corresponding to three RD series FE models, the values of which are also approximately equal. Accordingly, it can be concluded that changing the inner diameter of through diaphragm, within a reasonable range, has little influence on the cyclic behavior of the joint.

**Figure 27:**
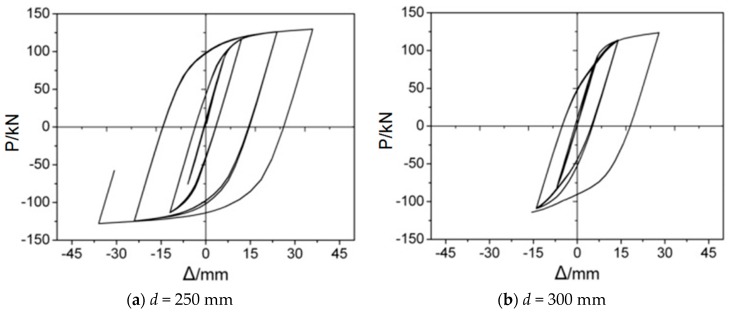
*P–Δ* curves of two joints.

As is well known, the larger the bore size, the easier for concrete pouring. However, if the value of *d* is over 250 mm, the FEM analysis will be terminated early. Here, 250 mm and 300 mm are employed as examples of the values of *d*, to illustrate this phenomenon. Figure 27 shows the corresponding analysis results. It is clearly seen that the FE analyses of both joints are not converged, and are terminated when the displacements of the beam end arrive at 36 mm, and 18 mm, respectively. The main reason for this phenomenon is that the effective area of through diaphragm inside the concrete is too small to transfer the stress of the beam to the core concrete, which causes several finite elements on the inside of through diaphragm, to violate shape warning limits during finite element analysis

With the adding cyclic displacement, the diaphragm stress will become higher, the crushes of core concrete develop quickly and accumulated in the swelling area, accelerating the process of the peeling between steel tube and concrete due to the stress concentration. Finally, the joint fails before reaching ultimate strength. Consequently, according to the simulation results from FE models, it could be concluded that the joint has good properties when *D*−*b_f_* ≤ *d* ≤ *b_d_*−*b_f_*, where *D* denotes column diameter and *b_d_* denotes outside diameter of diaphragm.

## 4. Orthogonal Experimental Design

In the last section, each parameter of the FE joint model was analyzed, and its effect on the mechanical property and seismic performance, was investigated as well. However, in practice, it is of great difficulty to evaluate which factors are dominant in affecting the joint performance via changing the value of a single parameter. Consequently, in this part, the orthogonal design is employed to comprehensively optimize the values of four parameters in FE joint model and to achieve the optimal design [22,23,24]. Previous studies have sufficiently proved the effectiveness of the orthogonal design in axial capacity analysis of rectangular/circular CFST columns [25,26]. Here three-level orthogonal arrays are used to understand the influence of four independent variables with three groups of values of each variable, as listed in Table 2. Four variables (parameters) include the RBS dimension (*a*, *b* and *c*) and the internal diameter of through diaphragm (*d*). The joint strength and energy dissipation coefficient are used as the evaluation indicators, where the joint strength is the ultimate loading of joint and the energy dissipation coefficient can be calculated by Equation (9). *K_i_* (*i* = 1, 2, 3) is the mean value of evaluation indicator for *i*th variable and *R_b_* denotes the error between maximum and minimum values of evaluation index. It can be found from the results in Table 2 that the influence on the strength of joint decreases in the order of *c* > *a* > *d* > *b* and the descending order sorted by the energy dissertation capacity of joint is *b* > *a* > *c* > *d*. The results from this orthogonal experimental design are able to provide the theoretical guidance for civil engineers to design the CFST column to RBS beam joint with through diaphragm for different purposes in the practical applications.

## 5. Conclusions

This paper has provided a comparative analysis on CFST circular column and RBS steel beam diaphragm-through joint in terms of stiffness degradation, load-displacement curves, the Von Mises stress distributions along beam and welding seam lengths, and energy dissipation. The relevant conclusions can be summarized as follows.

(1) To avoid excessive deterioration of steel at the reduced beam section, caused by heat affected zone of the joint, the range from the diaphragm fringe to the cut start (*a*) should be large enough. However, if the value of *a* is too large, the effect of RBS on the welding stress will be decreased. As a result, the optimal value of a should be between 0.5*b_f_* and 0.65*b_f_*, which not only can avoid steel deterioration at reduced beam section, but can also protect the joint from brittle failure.
(2) The cut length (*b*) has little influence on the stiffness, strength and load capacity of the composite joint.
(3) The addition of the cut depth (*c*) will result in obvious reduction of the stiffness, strength and load capacity of the composite joint. Hence, in the consideration of the stress distribution at key parts of the joint, the optimal value of *c* should be between 0.2*b_f_* and 0.25*b_f_*.
(4) The range of inner diameter of through diaphragm (*d*) should be *D*−*b_f_* ≤ *d* ≤ *b_d_*−*b_f_*, which can meet the load requirement and guarantee the pouring quality of the concrete in the steel tube meanwhile.
(5) The orthogonal design demonstrates that the importance order for the effect of each parameter on the composite joint strength should be cut depth (*c*), the range from the diaphragm fringe to the cut start (*a*), inner diameter of through diaphragm (*d*) and cut length (*b*), and the importance order for the effect of each parameter on the energy dissipation of the composite joint should be inner diameter of through diaphragm (*d*), cut depth (*c*), cut length (*b*) and the range from the diaphragm fringe to the cut start (*a*). The optimal combination of the parameters can be found using this orthogonal analysis

This research mainly focuses on the numerical analysis of the circular CFST column and RBS steel beam joint, even though experimental data from published literatures have been utilized to validate the capacity of the developed FE model of the joint. In the future, more specimens, with optimal parameter combinations, will be designed and tested in the laboratory, in order to further validate the effectiveness of the model in terms of strength and energy capacity prediction.

## Figures and Tables

**Table 1:** Finite element (FE) models dimensions and measured results.

Model No.	*a*/mm	*b*/mm	*c*/mm	*d*/mm	Distance (mm)	Stress (MPa)	*β*
RR	80	280	35	160	228.0	405.3	0.4250
RA1	60	280	35	160	199.5	403.4	0.4233
RA2	100	280	35	160	242.3	406.3	0.4250
RA3	120	280	35	160	240.0	407.1	0.4253
RB1	80	310	35	160	235.1	401.2	0.4267
RB2	80	340	35	160	242.3	398.6	0.4277
RB3	80	360	35	160	256.5	397.1	0.4280
RC1	80	280	30	160	228.0	396.1	0.4225
RC2	80	280	40	160	228.0	413.8	0.4260
RC3	80	280	45	160	228.0	421.3	0.4269
RD1	80	280	35	200	228.0	405.3	0.4396
RD2	80	280	35	220	228.0	405.3	0.4381
RD3	80	280	35	240	228.0	405.3	0.4372

**Table 2:** Orthogonal design (L_9_3^4^).

Specimens No.		*a*	*b*	*c*	*d*	Strength	Energy Dissipation Coefficient	
		(mm)	(mm)	(mm)	(mm)	(10^5^ N)		
1		A1(60)	B1(280)	C1(30)	D1(200)	1.37339	2.77532	
2		A1(60)	B2(310)	C2(35)	D2(220)	1.31558	2.77994	
3		A1(60)	B3(340)	C3(40)	D3(240)	1.25134	2.73849	
4		A2(80)	B1(280)	C2(35)	D3(240)	1.33054	2.68503	
5		A2(80)	B2(310)	C3(40)	D1(200)	1.27376	2.80486	
6		A2(80)	B3(340)	C1(30)	D2(220)	1.39915	2.77239	
7		A3(100)	B1(280)	C3(40)	D2(220)	1.29362	2.74388	
8		A3(100)	B2(310)	C1(30)	D3(240)	1.40847	2.63438	
9		A3(100)	B3(340)	C2(35)	D1(200)	1.36525	2.80684	
	Strength				Energy dissipation			
	*a*	*b*	*c*	*d*	*a*	*b*	*c*	*d*
*K*_1_	1.31344	1.33252	1.39367	1.33747	2.76458	2.73474	2.72736	2.79567
*K*_2_	1.33448	1.33260	1.33712	1.33612	2.75409	2.73973	2.75727	2.76540
*K*_3_	1.35578	1.33858	1.27291	1.33012	2.72837	2.77257	2.76241	2.78597
*R*_b_	0.04234	0.00606	0.12076	0.00735	0.03621	0.03783	0.03505	0.03027
Importance	*c > a > d > b*				*b > a > c > d*			
